# Supplementary material for: The Association of Asthma with Anxiety, Depression, and Mild Cognitive Impairment among Middle-Aged and Elderly Individuals in Saudi Arabia
Source: Behav Sci (Basel). 2023 Oct 16;13(10):842. doi: 10.3390/bs13100842 (PMC10604786; doi:10.3390/bs13100842)
Supplement: Supplementary file 1 [file behavsci-13-00842-s001.zip › behavsci-2577090-supplementary.pdf]

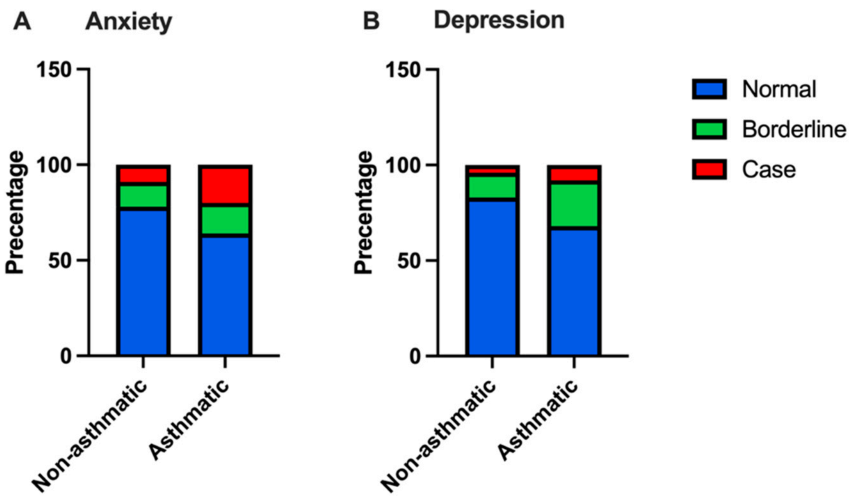

Supplementary Figure S1: The prevalence of (A) anxiety and (B) depression among asthmatic compared to non-asthmatic individuals.

Supplementary Table S1: Mean scores (± SD) of anxiety, depression, and cognition using study questionnaires and ANOVA p-values without controlling for covariates.

Variables	Asthmatic (n=84)	Non-asthmatic (n=159)	Total (n=243)	P-value [b]
**Anxiety**	6.2 ± 4.7	4.7 ± 3.5	5.2 ± 4.0	0.004
**Depression**	5.2± 3.9	4.1 ± 3.3	4.5 ± 3.6	0.01
**MoCA Test[a]**	23.7 ± 5.0	25.5 ± 3.7	24.9 ± 4.2	0.004

Supplementary Table S2: Multinomial logistic regression on the association between asthma and categories of anxiety and depression, while controlling for covariates.

Variables	Anxiety				Depression			
	Borderline		Case		Borderline		Case	
	OR (CI 95%)	P-value	OR (CI 95%)	P-value	OR (CI 95%)	P-value	OR (CI 95%)	P-value
**Age**	0.99 (0.95-1.03)	0.7	0.97 (0.93-1.02)	0.3	0.99 (0.95-1.03)	0.6	0.98 (0.92-1.05)	0.6
**Gender (Female) [a]**	1.39 (0.52-3.73)	0.5	3.57 (0.79-16.1)	0.1	-	-	-	-
**BMI**	1.02 (0.95-1.09)	0.6	1.05 (0.99-1.13)	0.09	1.01 (0.95-1.07)	0.7	**1.09 (1.00-1.20)**	**0.05**
**Asthma**	1.46 (0.65-3.27)	0.3	2.02 (0.87-4.68)	0.1	**2.10 (1.00-4.43)**	**0.05**	1.72 (0.50-5.93)	0.4

a Gender was removed from the model for depression because some categories had only one gender

Supplementary Table S3: Binomial logistic regression on the association between asthma and anxiety and depression

Variables	Anxiety [a]		Depression [a]	
	OR (CI 95%)	P-value	OR (CI 95%)	P-value
**Age**	0.98 (0.95-1.02)	0.3	0.99 (0.95- 1.03)	0.5
**Gender (Female)**	1.39 (0.83-4.51)	0.1	1.12 (0.49-2.61)	0.8
**BMI**	1.03 (0.98-1.09)	0.2	1.03 (0.98-1.09)	0.2
**Asthma**	1.70 (0.90- 3.19)	0.1	**1.99 (1.02- 3.90)**	**0.05**

a Borderline and cases were combined into one category.

Supplementary Table S4: Multinomial logistic regression on the association between asthma severity and anxiety and depression and MCI while controlling for covariates.

Variables	Anxiety/Depression				Cognition	
	Anxiety or Depression		Anxiety & Depression		Mild cognitive impairment	
	OR (CI 95%)	P-value	OR (CI 95%)	P-value	OR (CI 95%)	P-value
**Gender (Female)**	0.46 (0.10-2.04)	0.3	1.35 (0.21- 8.65)	0.7	0.40 (0.09- 1.74)	0.2
**BMI**	0.98 (0.88-1.07)	0.6	1.03 (0.94-1.13)	0.4	1.03 (0.95-1.12)	0.4
**Age**	0.94 (0.88-1.01)	0.1	0.97 (0.91-1.03)	0.4	**1.07 (1.01-1.13)**	**0.02**
**Asthma severity (Mild)**						
**Sever**	2.41 (0.38-15.8)	0.3	**10.8 (1.81-64.6)**	**0.009**	2.24 (0.42-11.8)	0.3
**Moderate**	0.89 (0.23-3.40)	0.9	1.63 (0.37-7.21)	0.5	1.02 (0.35-3.00)	0.9
